# Male-male competition and female choice are differentially affected by male call acoustics in the serrate-legged small treefrog, * Kurixalus odontotarsus*

**DOI:** 10.7717/peerj.3980

**Published:** 2017-10-31

**Authors:** Bicheng Zhu, Jichao Wang, Longhui Zhao, Qinghua Chen, Zhixin Sun, Yue Yang, Steven E. Brauth, Yezhong Tang, Jianguo Cui

**Affiliations:** 1 Department of Herpetology, Chengdu Institute of Biology, Chinese Academy of Sciences, Chengdu, China; 2 University of Chinese Academy of Sciences, Beijing, China; 3 Department of Biology, Hainan Normal University, Haikou, Hainan, China; 4 Ministry of Environmental Protection, South China Institute of Environmental Sciences, Guangzhou, Guangdong, China; 5 Department of Psychology, University of Maryland, College Park, MD, USA

**Keywords:** Signal evolution, Suppression call, Male-male competition, Female choice, Sexual dimorphism, *Kurixalus odontotarsus*

## Abstract

**Background:**

The evolution of exaggerated vocal signals in anuran species is an important topic. Males and females have both evolved the ability to discriminate communication sounds. However, the nature of sexual dimorphism in cognition and sensory discrimination and in the evolution and limitation of sexual signal exaggeration remain relatively unexplored.

**Methods:**

In the present study, we used male calls of varied complexity in the serrate-legged small treefrog, * Kurixalus odontotarsus*, as probes to investigate how both sexes respond to variations in call complexity and how sex differences in signal discrimination play a role in the evolution of sexual signal exaggeration. The compound calls of male* K. odontotarsus* consist of a series of one or more harmonic notes (A notes) which may be followed by one or more short broadband notes (B notes).

**Results:**

Male playback experiments and female phonotaxis tests showed that increasing the number of A notes in stimulus calls elicits increased numbers of response calls by males and increases the attractiveness of the stimulus calls to females. The addition of B notes, however, reduces male calling responses. Moreover, call stimuli which contain only B notes suppress spontaneous male calling responses. Phonotaxis experiments show that females prefer calls with greater numbers of A notes and calls containing both A notes and B notes, but do not prefer calls with only B notes.

**Discussion:**

Male-male competition and female choice appear to have played different roles in the evolution and limitation of signal complexity in *K. odontotarsus*. These results provide new insights into how exaggerated compound signals evolve and how signal complexity may be limited in anurans.

## Introduction

In some anuran species, males produce a single type of sexual signal, while in others highly complex and graded sexual signals have evolved which enable males to attract females and compete with rivals (Narins & Capranica, 1978; Greer & Wells, 1980; Rand & Ryan, 1981; Schwartz & Wells, 1984; Schwartz & Wells, 1985; Berglund, Bisazza & Pilastro, 1996; Zhu et al., 2016a; Zhu et al., 2016b). For example, males in two Panamanian species, *Dendropsophus ebraccatus* and *D. microcephalus*, which exhibit graded variation in both advertisement and aggressive calls, can adjust the relative aggressiveness and attractiveness of their calls in a graded fashion, depending on the proximity of their opponents (Wells & Schwartz, 1984; Wells & Bard, 1987; Wells, 1989). The evolution of such exaggerated vocal signals is believed to result from sexual selection (Andersson, 1994). In addition, predation pressure, working memory capacity, perceptual biases and choosier cognitive biases have also been found to foster the evolution of signal exaggeration (Stoddard, 1999; Akre & Ryan, 2010; Cui et al., 2016). In contrast, predation and parasitism pressures, as well as limitations in female cognitive processes can limit the evolution of sexual signal elaboration (Ryan, Tuttle & Rand, 1982; Bernal & Ryan, 2006; Akre et al., 2011).

Sexual selection differs in its form and consequences with regard to males and females. Although males and females have both evolved the ability to discriminate communication sounds, females generally exhibit greater selectivity than males (Forstmeier et al., 2014; Zhu et al., 2016a). However, the nature of sexual dimorphism in cognition and sensory discrimination and in the evolution and limitation of sexual signal exaggeration remain relatively unexplored.

The present study investigated the role of the acoustics of male calls in male-male competition and female choice in a tropical anuran species, the serrate-legged small treefrog, *Kurixalus odontotarsus* (Frost, 2017). Male *K. odontotarsus* produce several different call types with graded complexity to communicate with intraspecific individuals during the breeding season (Zhu et al., 2016a). However, the functions of these complex calls are not fully understood and, in particular, it is not known if each component of the male’s compound call affects males and females differently. This species also offers an opportunity to investigate how exaggerated characteristics relevant to mating can evolve.

In the present study, we used the serrate-legged small treefrog *K. odontotarsus* to investigate (1) how both sexes respond to variation in call complexity, (2) the role of sex differences in sensory discrimination in the evolution and limitation of sexual signal exaggeration. Female phonotaxis tests and male evoked vocal response experiments using calls of different complexity were used in these experiments. Male calls are composed of two different note types whose numbers vary between calls in *K. odontotarsus*.

## Materials & Methods

### Vocalization recordings

The recordings were conducted in the Mt. Diaoluo National Nature Reserve in Hainan, China (18.44°N, 109.52°E, elevation of 933 m a.s.l.) from April to May, 2014. Each male was recorded for 3 min, using a directional microphone (ME66 with K6 power module, Sennheiser, Germany) connected to a digital recorder (PMD 660, 16 bit, 44.1 kHz, Marantz, Japan), from 20:00 to 24:00 h (Temperature: 20.4 ± 0.52 °C, Relative Humidity: 94.9 ± 3.96%). The calls of 62 males were recorded in their original habitat in order to provide a large sample of communication sounds for acoustic analysis. Prior to being returned to the habitat, each male was given a unique toe-clip number to prevent repeated recording.

### Acoustic stimuli

**Figure 1:**
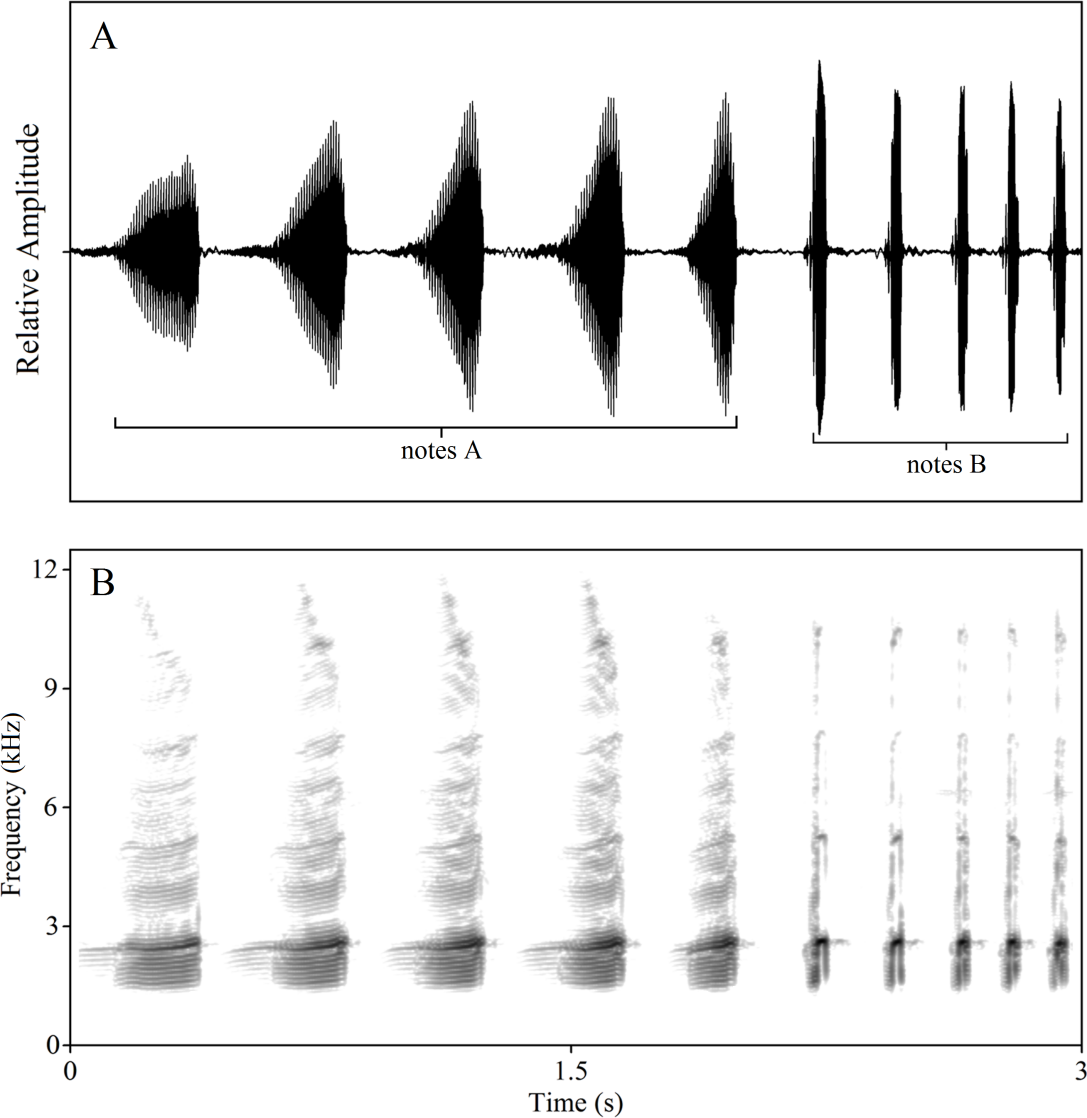
Amplitude-modulated waveforms (A) and spectrograms (B) of male compound call “5A5B”. The FFT (fast Fourier transform) frame is 1,024.

In a previous study, we found that most of the complex calls of *K. odontotarsus* consist of two types of notes, note A and note B, with differing note numbers per call. Note A is a harmonic sound spanning a wide frequency band while note B is a shorter broadband sound. Note B is much shorter in duration than note A and does not exhibit the distinctive pulsed structure of note A (Zhu et al., 2016a; Köhler et al., 2017). Males typically produce three kinds of calls: A-note calls, B-note calls and compound calls containing both kinds of notes (Fig. 1, also see Zhu et al., 2016a). Of interest is whether these call types serve different behavioral functions. In order to evaluate the behavioral functions and likely selection pressures acting on the evolution of these call types, three classes of stimuli were constructed: (1) A calls consisting of 1, 3 or 5 notes (1A, 3A, 5A), (2) B calls consisting of 1, 3 or 5 notes (1B, 3B, 5B), (3) compound calls consisting of 5 notes A and 2 or 5 notes B (5A2B, 5A5B). All stimulus types used in the experiments were constructed using natural calls and are listed in Table 1.

To minimize pseudoreplication effects, five exemplars of each call type, derived from five different calling males were used. Each test stimulus combination was labeled with a numerical code of the form “X^y^” in which “X” represented the stimulation types (i.e., number of A and B notes in the call pair used in the test) and “y” represented the male number. For example, the number “8^5^” in the female phonotaxis tests stands for the stimulus pair “5A2B” versus “5A5B” (see Table 1), derived from the calls of the fifth calling male. To control for potential order effects, all test stimuli were randomly broadcast. Randomized call orders were generated using the Randbetween function in Excel. The test stimuli were broadcast with a 5-s interstimulus intervals (approximately equal to the mean intercall intervals in the chorus). To minimize bias, observers were blind to the experimental conditions in use during recording and analysis of all female phonotaxis and male response data. Prior to returning the frogs to their original habitat, the subjects were given a unique toe-clip number to avoid being retested.

**Table 1:** The acoustic stimuli used in the female phonotaxis tests and male playback experiments.

	Female phonotaxis test		
Test number	Alternative 1 stimulus	Alternative 2 stimulus	Male playback tests stimulus
1	5A^1,2,3,4,5^	Silence	5A^1,2,3,4,5^
2	5B^1,2,3,4,5^	Silence	5B^1,2,3,4,5^
3	5A^1,2,3,4,5^	5B^1,2,3,4,5^	3A^1,2,3,4,5^
4	2A^1,2,3,4,5^	5A^1,2,3,4,5^	3B^1,2,3,4,5^
5	2B^1,2,3,4,5^	5B^1,2,3,4,5^	1A^1,2,3,4,5^
6	5A^1,2,3,4,5^	5A2B^1,2,3,4,5^	1B^1,2,3,4,5^
7	5A^1,2,3,4,5^	5A5B^1,2,3,4,5^	5A2B^1,2,3,4,5^
8	5A2B^1,2,3,4,5^	5A5B^1,2,3,4,5^	5A5B^1,2,3,4,5^

**Notes.**

5A call containing five A notes 5B call containing five B notes 5A2B call containing five A notes followed by two B notes

Other abbreviations are labeled accordingly.

The superscript numbers (1, 2, 3, 4, 5) of each stimulus represent the calling male from which each stimulus was derived from five different calling males.

### Female phonotaxis experiments

Female phonotaxis tests were conducted in the Mt. Diaoluo National Nature Reserve in Hainan, China (18.44°N, 109.52°E, elevation of 933 m a.s.l.) from April to June, 2014. A total of 65 gravid females were utilized in the phonotaxis tests. We performed standard two-speaker phonotaxis tests offering females a choice involving eight stimuli pairs (Table 1), which were presented to females between 20:00 h to 23:00 h (Temperature: 21.3 ± 0.35 °C, Relative Humidity: 93.5 ± 3.12%). To avoid experimental fatigue, each female on average was allowed a 3-minute break between consecutive tests.

Each female was placed in the center of a sound-attenuating chamber (inside dimensions, *L* ×*W* × *H*: 250 ×150 × 150 cm). We broadcast the stimulus pairs using two portable field speakers (SME-AFS, Saul Mineroff Electronics, Elmont, NY, USA) placed equidistant from the opposite sides of the chamber. The distance between the two speakers was 220 cm. To control for potential side bias, we broadcast the test stimuli antiphonally such that the “fast” root-mean-square amplitude of each test call was 80 dB SPL (re 20 µPa, A-weighted, the approximate SPL of a natural call at the same distance) and alternated the presentation of each stimulus pair between the right and left loudspeaker after each test. The sound pressure levels (SPLs) of each test call were measured with a sound level meter (AWA 6291, Hangzhou Aihua Instruments Co., Zhejiang, China) at the release location of the subject. We scored a choice when the female approached within 10 cm of either speaker without simply following the wall. If a female spent more than 10 min roaming the arena without approaching a speaker, she was given a second chance at the end of the tests after a 10 min break. If the female still failed to approach a speaker, no choice was recorded. We observed the behavior of the females on a monitor using a wide-angle lens video system with an infrared light source (also see Zhu et al., 2016b). The frogs were returned that night to their original habitat after the tests were completed. Prior to being returned to their original habitat, the subjects were given a unique toe-clip number to avoid being retested.

### Male evoked vocal response experiments

Male evoked vocal response experiments were recorded at the same study site, from April to July, 2014. A total of 60 males were utilized in the playback tests. We found that male serrate-legged small treefrogs do not respond to playbacks in a sound-attenuating chamber. For this reason calling males were captured at breeding sites and brought to a field test site with an environment similar to the breeding sites where no nearby males were calling. Males used in the evoked vocal response experiments were tested with eight stimuli (Table 1), which were presented to males between 21:00 h to 24:00 h (Temperature: 20.9 ± 0.72 °C, Relative Humidity: 97.4 ± 3.58%). Each male subject was placed on the shrubbery. The playback stimuli were broadcast from a portable field speaker (SME-AFS, Saul Mineroff Electronics, Elmont, NY, USA) placed 1 m from the shrubbery (the approximate distance of two nearby males in their natural environment). Each stimulus was played for three minutes. The response calls of males before, during and after playback were recorded for three minutes respectively, using an Aigo R5518 recorder with an internal microphone (Aigo Digital Technology Co. Ltd., Beijing, China). The “fast” root-mean-square amplitude of the broadcast test stimuli at the release position was 80 dB SPL (re 20 µPa, A-weighted, the approximate SPL of a natural call at the same distance). The sound pressure levels (SPLs) of each test call were measured with a sound level meter (AWA 6291, Hangzhou Aihua Instruments Co.). The frogs were returned that night to their original habitat after the tests were completed. Prior to being returned to their original habitat, the subjects were given a unique toe-clip number to avoid being retested.

### Analysis and statistics

The sonograms of calls were generated using free PRAAT software (Boersma, 2002). Data were statistically analyzed and graphs created using Sigmaplot 11.0 software (Systat Software Inc., Chicago, USA). The Chi-square test was used to evaluate the female phonotaxis results. The effects of stimulus type and playback time (before, during and after playback) on male evoked vocal responses were analyzed using Two Way Repeated Measures ANOVA (Two Factor Repetition). If a statistically significant difference was found, a multiple comparison procedure (Holm-Sidak method) was used to evaluate the differences among groups. All data were expressed as Mean ± SD, and *p* < 0.05 was considered to be statistically significant.

### Ethics note

All applicable international, national, and/or institutional guidelines for the care and use of animals were followed. All procedures performed in studies involving animals were approved by the Animal Care and Use Committee of Chengdu Institute of Biology, CAS (CIB2014031008). This work was conducted with the permission of the Management Office of the Mt. Diaoluo Nature Reserve.

## Results

### Analysis of call complexity

Through long-term field observations, we have found that contests in this species generally begin with an exchange of note A calls, then involve exchanges of note B calls and, in some cases, physical combat. Male serrate-legged small treefrogs produce calls of varied complexity. Males can produce a compound call, which consists of a series of multi-harmonic A notes with one or several B notes (Fig. 1). Males, usually, can add one to five B notes to several A notes. We referred to the measure of signal complexity in túngara frogs (Bernal et al., 2009; Akre et al., 2011) and described the call complexity of *K. odontotarsus*. The complexity of vocal signals is thus manifest at two levels: the number of notes and note types. Typically, calls contain 3.8 ± 0.9 (mean ± sd.) A notes. Calls with 3–5 notes are more complex than those containing only one or two notes (i.e., 5A vs. 2A). The compound calls containing two note types (such as the call shown in Fig. 1 with five A notes followed by five B notes, labeled 5A5B) may be regarded as more complex than those containing only one note type (i.e., 5A2B vs. 5A). In the field, more than half of the calls we recorded contained A notes followed by B notes. In all compound calls (*n* = 96), 85.6% have one or two B notes, while more than 96.7% have 1–3 B notes. In summary, male serrate-legged small treefrogs can produce calls of varying complexity consisting of varying numbers of A notes and B notes in response to potential rivals and to attract mates.

### Female phonotaxis responses

**Figure 2:**
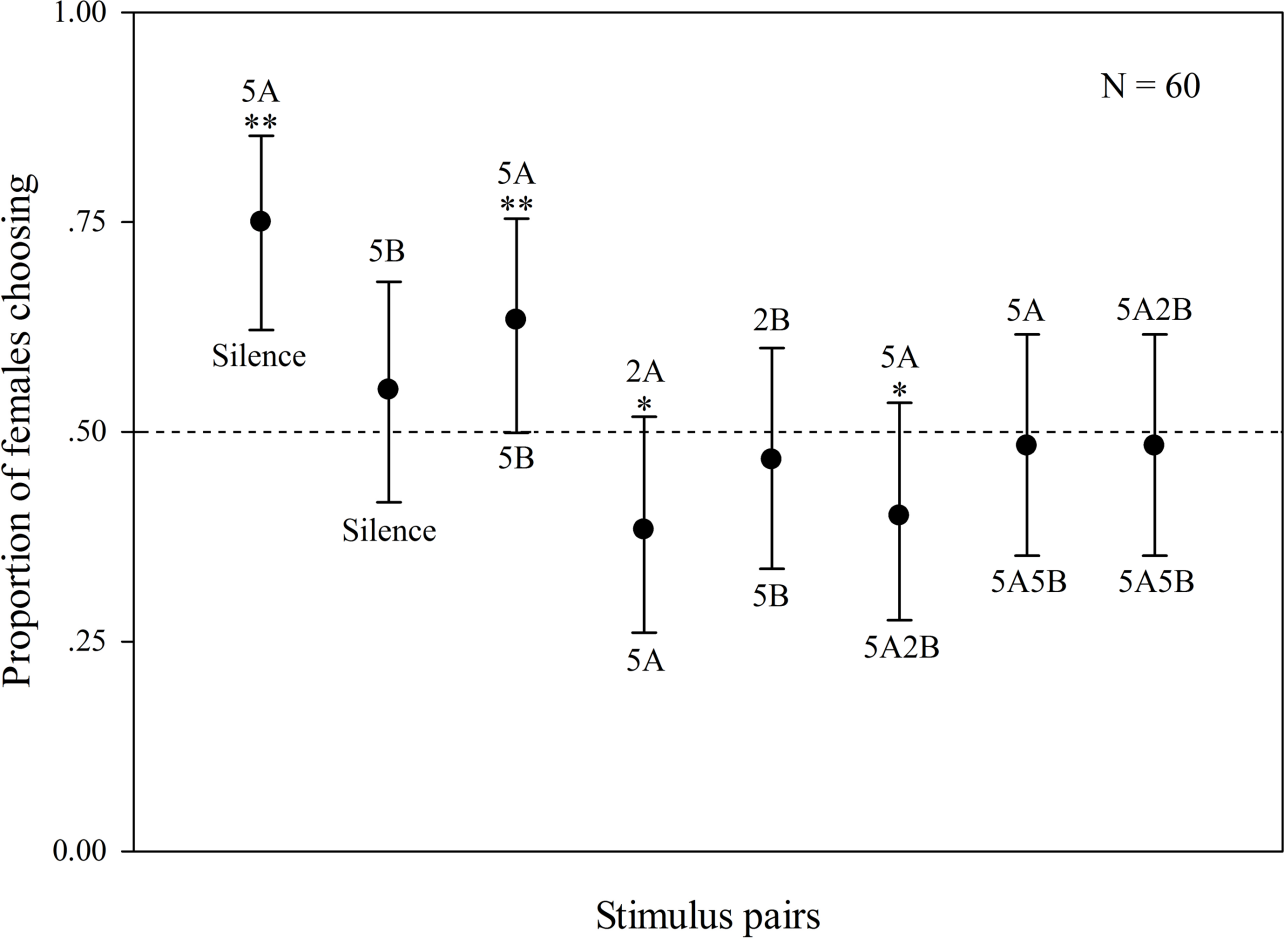
Results of female phonotaxis tests for *K. odontotarsus*. The different playback stimuli are labeled according to the numbers of each note type contained in each stimulus and include: 5A, 5B, 3A, 3B, 1A, 1B, 5A2B, 5A5B. The dashed line indicates the proportion of females choosing one of the other alternatives based on chance (50%). Bars are 95% confidence intervals. ^∗^*p* < 0.05, ^∗∗^*p* < 0.01, Chi-square test.

The results are shown in Fig. 2. Some females did not behaviorally indicate a choice (5 females). When “5A” and silence were played back alternately, 75% of the females preferred “5A” (*χ*^2^ = 25.000, *p* < 0.001, *n* = 60); when “5A” and “5B” were played back alternately, 63.3% of the females preferred “5A” (*χ*^2^ = 6.760, *p* = 0.009, *n* = 60). These results demonstrate that note A is a more attractive sexual signal to females than note B. When the more complex “5A” and simpler “2A” were played back, 61.7% of the females preferred “5A” (*χ*^2^ = 5.760, *p* = 0.016, *n* = 60). There was no significant difference between female responsiveness to “5B” and “silence” (*χ*^2^ = 1.000, *p* = 0.317, *n* = 60) as well as to “5B” and “2B” (*χ*^2^ = 0.360, *p* = 0.549, *n* = 60) consistent with the idea that females are not responsive to note B alone. In contrast 60% of the females preferred compound call “5A2B” to “5A” (*χ*^2^ = 4.000, *p* = 0.046, *n* = 60). However, there were no significant differences between female responses to call “5A2B” and call “5A5B” (*χ*^2^ = 0.160, *p* = 0.689, *n* = 60, Fig. 2).

### Male evoked vocal responses

A total of 4,320 min of calling was recorded. We analyzed the total number of notes, notes/call, maximum number of A notes and the number of compound calls in male evoked vocal responses. There are significant differences in male evoked vocal responses between stimuli (Notes: *F*_7,59_ = 10.923, *p* < 0.001; Maximum number of notes A: *F*_7,59_ = 6.650, *p* < 0.001; Two Way Repeated Measures ANOVA) and playback time (Notes: *F*_2,59_ = 41.238, *p* < 0.001; Maximum number of notes A: *F*_2,59_ = 21.878, *p* < 0.001; Two Way Repeated Measures ANOVA). There is also a statistically significant interaction effect between playback time and stimuli (Playback time × Sound interaction effect, Notes: *F*_2,7_ = 11.005, *p* < 0.001; Maximum number of notes A: *F*_2,7_ = 8.020, *p* < 0.001; Two Way Repeated Measures ANOVA).

**Figure 3:**
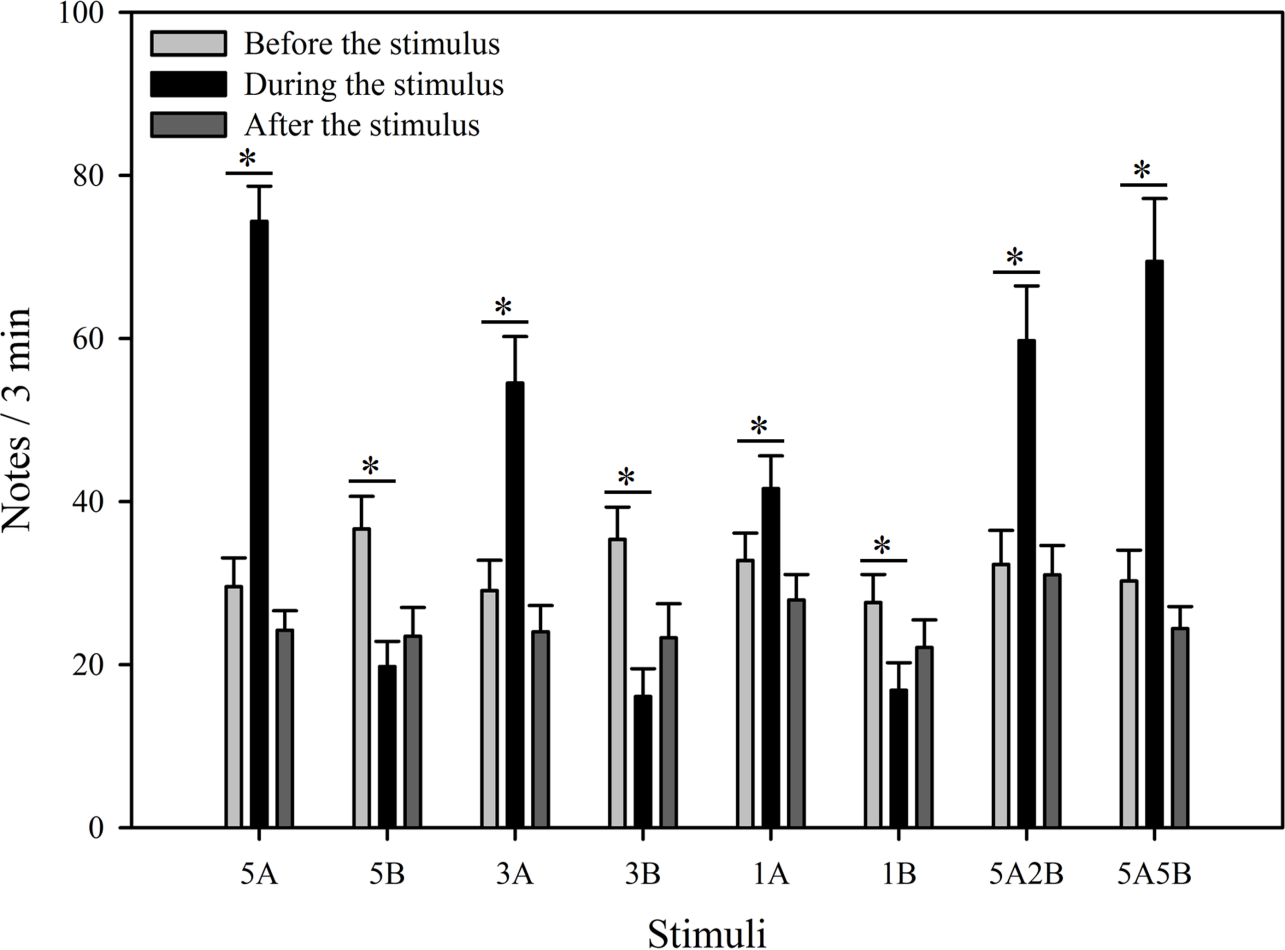
Male *K. odontotarsus* evoked vocal responses: the total number of notes produced in response to the stimuli. The different playback stimuli are labeled according to the numbers of each note type contained in each stimulus and include: 5A, 5B, 3A, 3B, 1A, 1B, 5A2B, 5A5B. We only show the statistical relationships between the period “during” which the stimuli were played back and “before” the stimuli were played back in the figure. The ∗ symbol represents a significant difference, Two Way Repeated Measures ANOVA and Holm-Sidak method.

The stimuli containing note A (5A, 3A, 1A, 5A2B, 5A5B) elicited more responses during the period the stimuli were played back than before the stimuli were played back. The total note number respectively increased by 151.7% with 5A, 87.5% with 3A, 26.9% with 1A, 85.0% with 5A2B, and 129.6% with 5A5B (Fig. 3). Interestingly, stimuli containing *only* note B (5B, 3B, 1B) *suppressed* male calling responses during the period the stimuli were played, such that the number of notes produced decreased by 46.0% with 5B, 54.5% with 3B, and 39.0% with 1B (Fig. 3). More statistical details are shown in Table 2. For all stimuli, male calling responses “before” playbacks were similar and not significantly different from those produced “after” (*p* = 0.4, Holm-Sidak method, Fig. 3). Responses to “5A” were greater than to “3A” (*p* = 0.001) during the playback, and stimulation using “3A” stimulation resulted in more responses than “1A” (*p* = 0.013) during the playback. Responses to “5A” were greater than to “5A2B” (*p* = 0.015) during the playback, and “5A5B” stimulation resulted in more responses than “5A2B” (*p* = 0.031) during the playback (Holm-Sidak method, Fig. 3, Table 3). Stimuli containing *only* note B (5B, 3B, 1B) which differed in the number of B notes did not yield statistically significant differences during the playback (1B vs. 3B: *p* = 0.899; 3B vs. 5B: *p* = 0.540; 1B vs. 5B: *p* = 0.628; Holm-Sidak method; Fig. 3; Table 3).

**Table 2:** The statistical results of male call responses during period the stimuli were played back compared to the period before the stimuli were played back.

		5A	5B	3A	3B	1A	1B	5A2B	5A5B
Total	Notes	<0.001	0.005	<0.001	0.001	0.023	0.029	<0.001	<0.001
	Notes/call	0.001	0.064	0.002	0.178	0.034	<0.001	0.002	<0.001
	Max. number of note A	<0.001	0.005	<0.001	<0.001	0.014	0.009	<0.001	<0.001
A note	notes	0.619	<0.001	0.427	<0.001	0.943	0.006	0.905	0.76
B note	notes	<0.001	0.785	<0.001	0.935	0.018	0.508	<0.001	<0.001

**Notes.**

5A call containing five A notes 5B call containing five B notes 5A2B call containing five A notes followed by two B notes

Other abbreviations are labeled accordingly. The numbers in each row are *P* values.

**Table 3:** The statistical differences between male call responses evoked by eight stimuli produced during the period the stimuli were played back.

		5B	3A	3B	1A	1B	5A2B	5A5B
Total notes	5A	<0.001	0.001	<0.001	<0.001	<0.001	0.015	0.415
	5B	–	<0.001	0.54	<0.001	0.628	<0.001	<0.001
	3A		–	<0.001	0.013	<0.001	0.387	0.013
	3B			–	<0.001	0.899	<0.001	<0.001
	1A				–	<0.001	0.003	<0.001
	1B					–	<0.001	<0.001
	5A2B						–	0.031
Note A	5A	0.014	0.528	0.004	0.784	<0.001	0.653	0.453
	5B	–	0.002	0.639	0.007	0.341	0.004	0.089
	3A		–	<0.001	0.722	<0.001	0.856	0.167
	3B			–	0.001	0.629	<0.001	0.03
	1A				–	<0.001	0.861	0.306
	1B					–	<0.001	0.008
	5A2B						–	0.23
Note B	5A	<0.001	<0.001	<0.001	<0.001	<0.001	<0.001	0.746
	5B	–	<0.001	0.716	0.026	0.656	<0.001	<0.001
	3A		–	<0.001	0.003	<0.001	0.124	<0.001
	3B			–	0.01	0.419	<0.001	<0.001
	1A				–	0.076	<0.001	<0.001
	1B					–	<0.001	<0.001
	5A2B						–	<0.001

**Notes.**

5A call containing five A notes 5B call containing five B notes 5A2B call containing five A notes followed by two B notes

Other abbreviations are labeled accordingly. The numbers in each row are *P* values.

**Figure 4:**
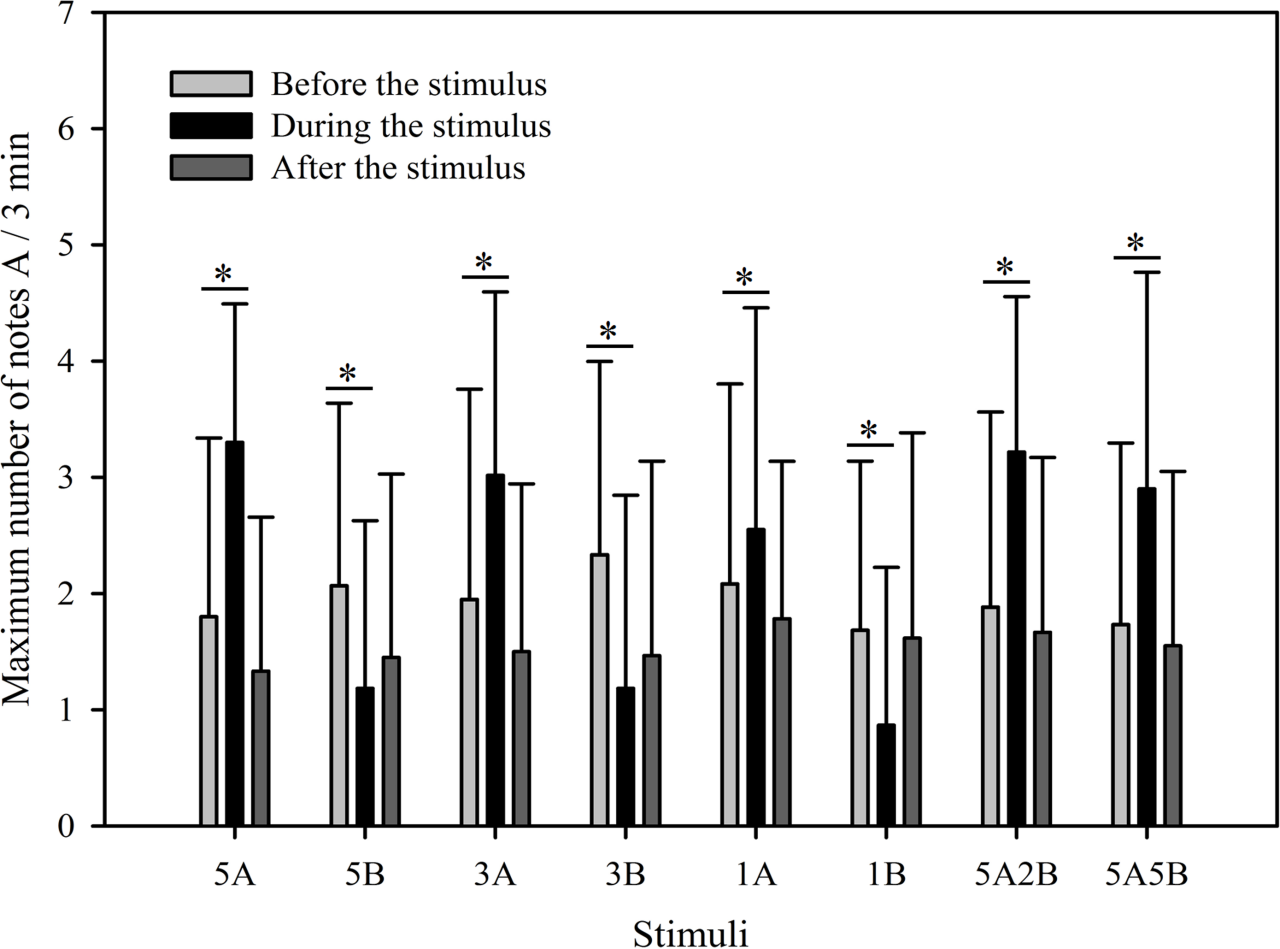
Male *K. odontotarsus* evoked vocal responses: the maximum number of note A produced in response to the stimuli. The * symbol represents a significant difference, Two Way Repeated Measures ANOVA and Holm-Sidak method.

The results show that the maximum number of notes A produced by males in response to all eight stimuli were similar as was the total note number. Male calling responses “during” playbacks differed significantly from those produced “before” (*p* < 0.001, Holm-Sidak method, Fig. 4). The stimuli containing note A elicited more complex calls during the period the stimuli were played back than before the stimuli were played back (5A: *p* < 0.001; 3A: *p* < 0.001; 1A: *p* = 0.014; 5A2B: *p* < 0.001; 5A5B: *p* < 0.001; Holm-Sidak method; Fig. 4; Table 2). However, stimuli containing *only* note B elicited fewer complex calls during the period the stimuli were played back than before the stimuli were played back (5B: *p* = 0.005; 3B: *p* < 0.001; 1B: *p* = 0.009; Holm-Sidak method; Fig. 4; Table 2).

**Figure 5:**
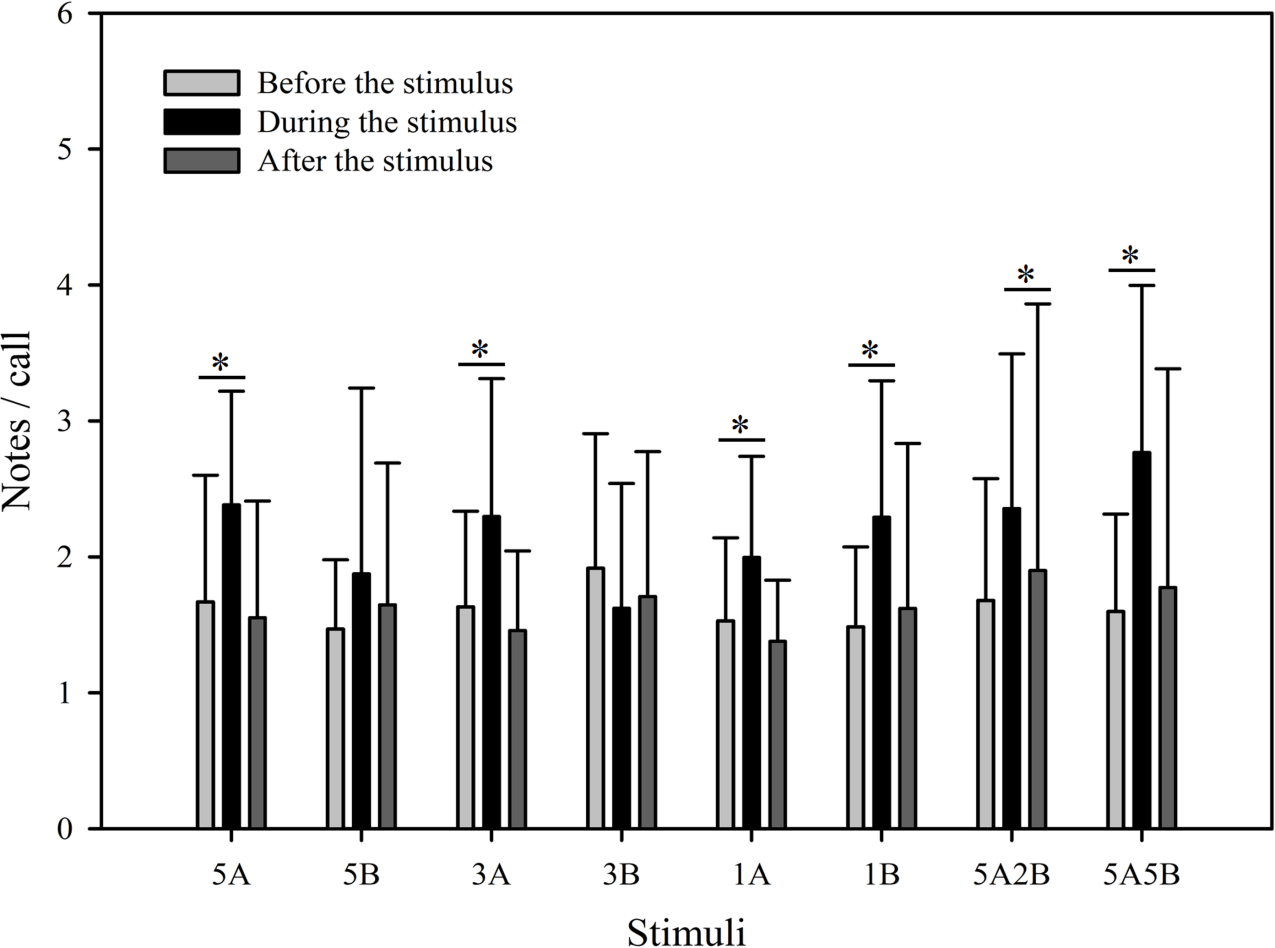
Male *K. odontotarsus* evoked vocal responses: the ratio of notes/call in response to the stimuli. The ∗ symbol represents a significant difference, Two Way Repeated Measures ANOVA and Holm-Sidak method.

Despite the fact that the number of notes/call which were evoked differed between stimuli (*F*_7,59_ = 2.561, *p* = 0.013, Two Way Repeated Measures ANOVA), the only stimuli containing note A yielding statistically significant differences during playback were “5A5B” and “1A”, and the only note B containing stimuli yielding significant differences during playback were “1B” and “3B” (5A5B vs. 1A: *p* < 0.001; 1B vs. 3B: *p* = 0.002; Holm-Sidak method; Fig. 5). The number of notes/call differed among playback periods (*F*_2,59_ = 36.307, *p* < 0.001, Two Way Repeated Measures ANOVA). Male calling responses “during” playbacks were significantly different from those produced “before” (*p* < 0.001) except for the stimulus “5B” (*p* = 0.064) and the stimulus “3B” (*p* = 0.178, Holm-Sidak method, Fig. 5, Table 2). For all stimuli, male calling responses “before” playbacks were similar and not significantly different from those produced “after” (*p* = 0.4, Holm-Sidak method, Fig. 5).

**Figure 6:**
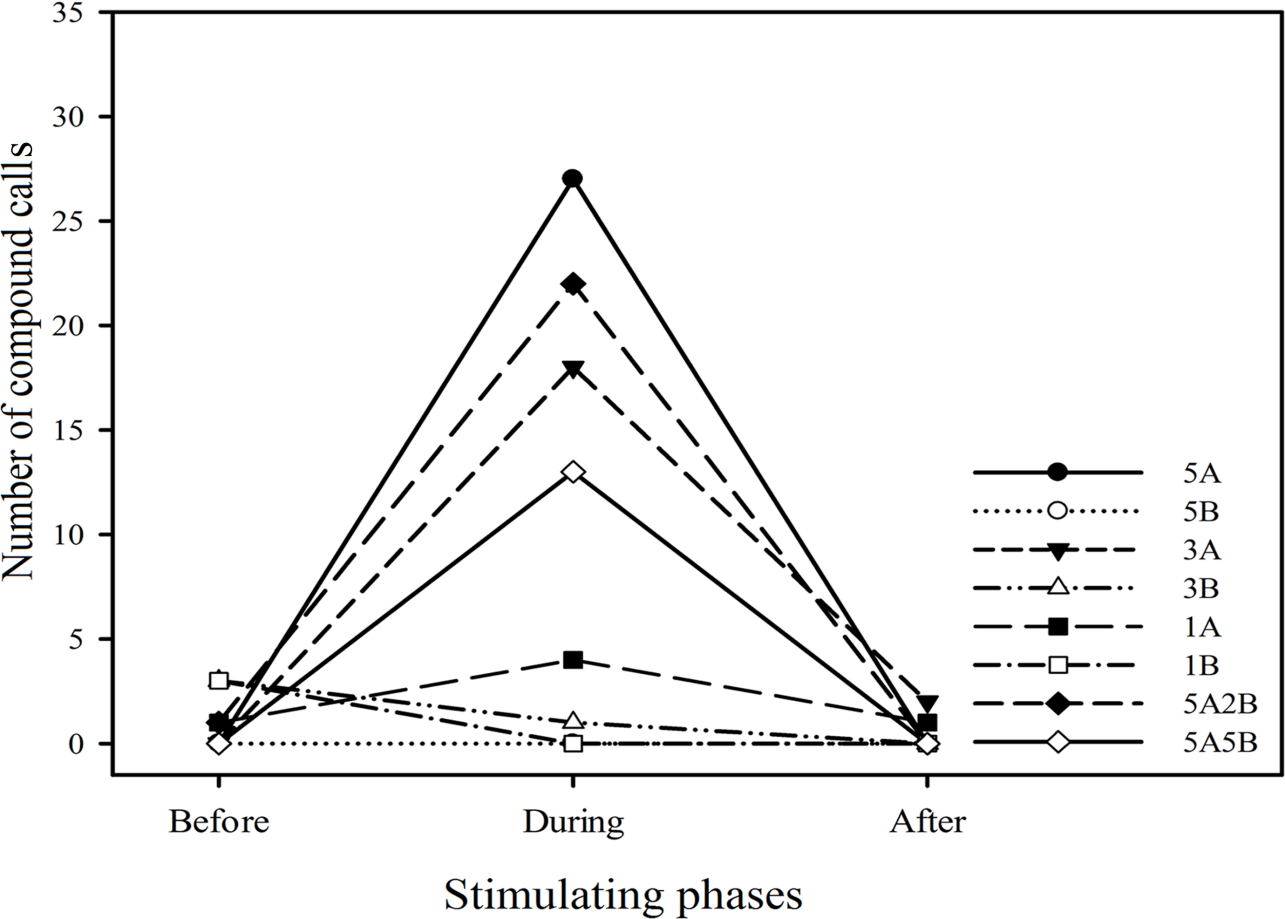
The total number of compound calls produced by males in response to each kind of playback stimulus. “Before”, “During” and “After” represent before, during and after the playback stimulus presentation period, respectively.

Twenty six (26) males (43.3%) emitted 96 compound calls which consisted of 4.33 ± 0.96 (mean ± sd.) A notes and 1.66 ± 0.93 (mean ± sd.) B notes. Males emitted more compound calls during the periods the stimuli containing note A (i.e., 5A, 3A, 1A, 5A2B, 5A5B) were played back (*n* = 84) than during the period before the stimuli were played back (*n* = 2). Notably males produced no compound calls or fewer compound calls during the period stimuli containing only note B (i.e., 5B, 3B, 1B) were played back (*n* = 1) compared to the period before these stimuli were played back (*n* = 6) (Fig. 6). In other words, playback stimuli containing note A but not stimuli only containing note B elicited increased numbers of compound calls.

**Figure 7:**
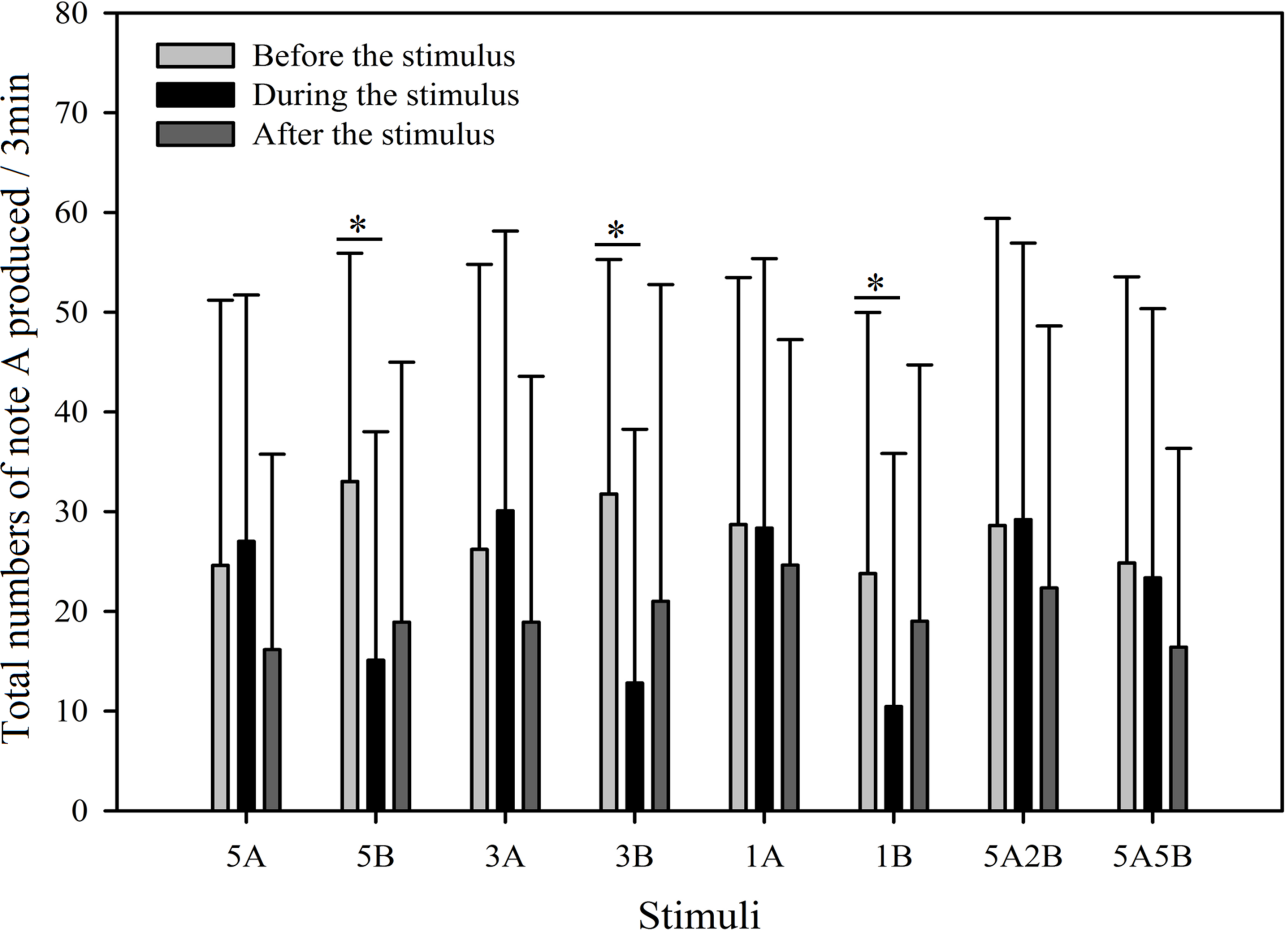
Male *K. odontotarsus* evoked vocal responses: the total number of notes A produced in response to the stimuli. The ∗ symbol represents a significant difference, Two Way Repeated Measures ANOVA and Holm-Sidak method.

**Figure 8:**
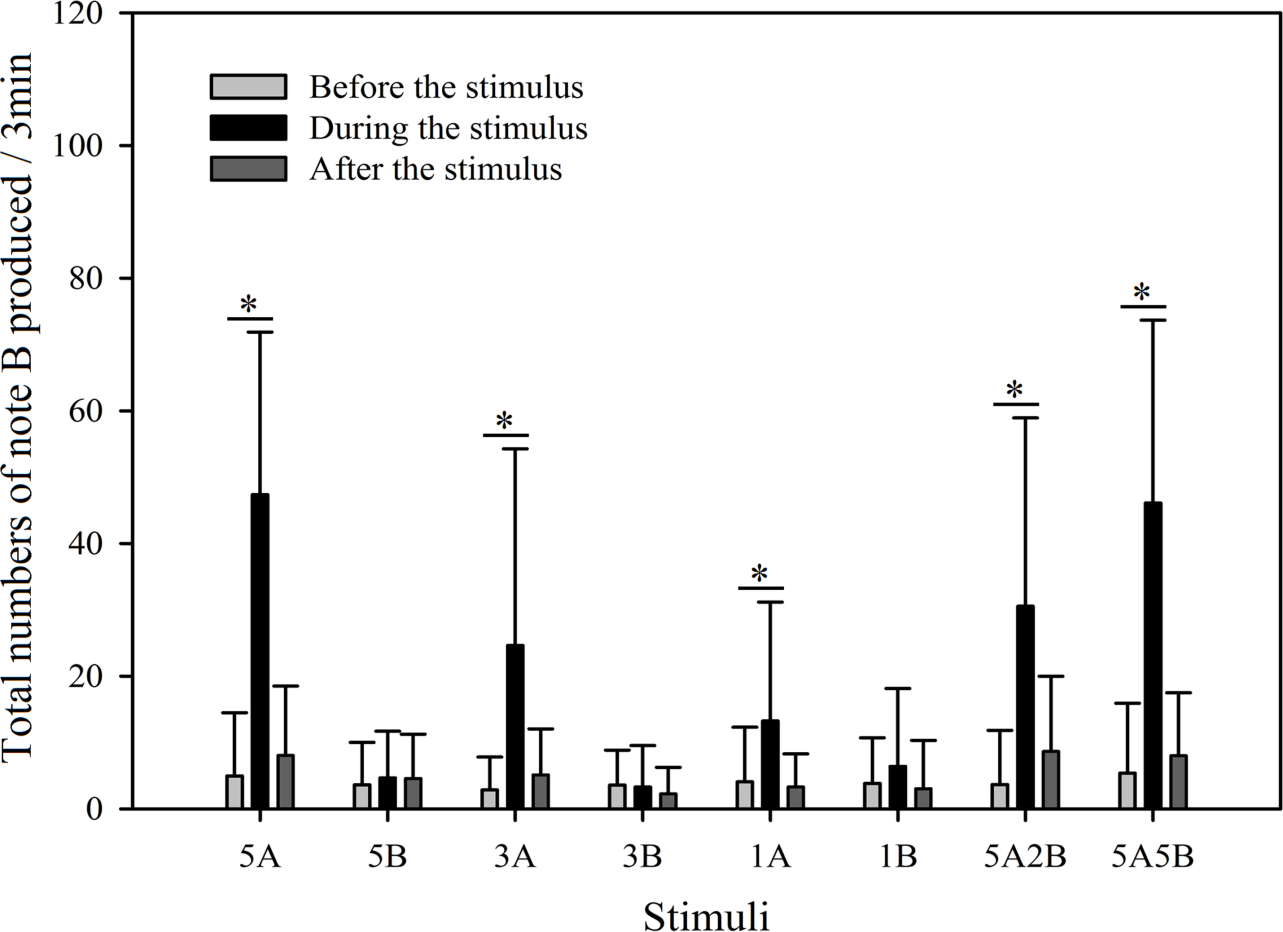
Male *K. odontotarsus* evoked vocal responses: the total number of notes B produced in response to the stimuli. The ∗ symbol represents a significant difference, Two Way Repeated Measures ANOVA and Holm-Sidak method.

In order to further investigate male call response patterns elicited by different playback stimuli, we counted the total number of notes A and notes B produced in all response calls. As can be seen in Figs. 7 and 8, total numbers of note A in calls produced and total numbers of note B in calls produced differed between stimuli (Note A: *F*_7,59_ = 2.484, *p* = 0.016; Note B: *F*_7,59_ = 19.540, *p* < 0.001; Two Way Repeated Measures ANOVA) and playback time (Note A: *F*_2,59_ = 11.564, *p* < 0.001; Note B: *F*_2,59_ = 108.358, *p* < 0.001; Two Way Repeated Measures ANOVA). We also found a statistically significant interaction effect between playback time and stimuli (Playback time × Sound interaction effect, Note A: *F*_2,7_ = 2.323, *p* = 0.004; Note B: *F*_2,7_ = 12.591, *p* < 0.001; Two Way Repeated Measures ANOVA). Stimuli containing *only* note B elicited fewer male calls containing note A during the period the stimuli were played back than before the stimuli were played back, such that the total numbers of note A produced decreased by 54.3% with 5B, 59.7% with 3B, and 56.0% with 1B (Holm-Sidak method, Fig. 7, Table 2). For all stimuli containing note A, male calls containing note A “before” playbacks were not significantly different from those produced “during” (5A: *p* = 0.619; 3A: *p* = 0.427; 1A: *p* = 0.943; 5A2B: *p* = 0.905; 5A5B: *p* = 0.760; Holm-Sidak method; Fig. 7; Table 2).

Stimuli containing A notes and stimuli containing only B notes did not yield statistically significant differences in the total numbers of note A produced during the playback (Fig. 7; Table 3). Furthermore, stimuli containing note A elicited more male calls containing note B during the period the stimuli were played back than before the stimuli were played back, such that the total number of B notes increased by 856.6% with 5A, 758.0% with 3A, 225.4% with 1A, 732.6% with 5A2B, and 753.7% with 5A5B (Holm-Sidak method, Fig. 8, Table 2). For all stimuli containing only note B, the numbers of male calls produced containing note B “before” playbacks were not significantly different from those produced “during” (5B: *p* = 0.78textit; 3B: *p* = 0.935; 1B: *p* = 0.508; Holm-Sidak method; Fig. 8; Table 2). As shown in Fig. 8, responses to “5A” were greater than to “3A” (*p* < 0.001) during the playback, and stimulation using “3A” resulted in more responses than “1A” (*p* = 0.003) during the playback (Table 3). Responses to “5A” were greater than to “5A2B” (*p* < 0.001) during the playback, and “5A5B” stimulation resulted in more responses than “5A2B” (*p* < 0.001) during the playback (Holm-Sidak method, Fig. 8, Table 3). Stimuli containing only note B did not yield statistically significant differences in the total numbers of note B produced during the playback (1B vs. 3B: *p* = 0.419; 3B vs. 5B:*p* = 0.716; 1B vs. 5B: *p* = 0.656; Holm-Sidak method ; Fig. 8; Table 3).

During the 6 min periods “before” and “after” the playback stimuli, male serrate-legged small treefrogs produced 15.60 ± 2.97 (mean ± sd.) calls containing note A, but produced 2.38 ± 1.30 (mean ± sd.) calls containing note B (Figs. 7 and 8).

## Discussion

The results of the phonotaxis experiments show that *K. odontotarsus* females preferred calls containing more A notes, indicating that note A is an attractive signal to females. Consistent with these female preferences, males produced more response calls when the playback stimuli included note A than were produced spontaneously before playback. These results indicate that note A as an advertisement call is the primary call type for female attraction, but also serves a role in male-male competition. While B notes were not particularly effective versus silence, 30–40% of the females chose B-note calls when pitted directly against A-note calls, suggesting that B notes are also attractive to females. The results of male evoked vocal response experiments show that *K. odontotarsus* males emitted more B notes during playbacks of stimuli which included note A than were produced before the playbacks, and emitted fewer notes A during the playbacks of stimuli which contained only note B than before the playbacks. These results imply that note B of male *K. odontotarsus* calls functions as a suppressor of competitors’ advertisement calls. Except for the test of A versus silence, the preferences demonstrated here are weak relative to those in the context of female choice in many studies of other frog species (Akre et al., 2011; Cui et al., 2016). However, failures to show preferences (or to selectively respond) do not necessarily reflect limitations of the auditory system.

When subjects perform in more than one test there is a possibility of carryover effects (Brooks, 2012). Each female in these experiments was provided only one opportunity to choose in a given test. Without test repetition, we can not directly detect whether an individual experiences carryover effects. For this reason we mitigated potential carryover effects by using a randomized sequence for broadcasting the stimulus tests and by providing each female a 3-minute break between consecutive tests.

Compound calls (several A notes + one or more B notes) function as advertisement calls, insofar as these calls both attract females and foster male-male competition. The addition of one or more B notes as an appendage in male compound calls increases the attractiveness of the call to females (i.e., 5A vs. 5A2B). For males, compound calls containing a few B notes tend to reduce vocal competition compared with calls containing only A notes (i.e., 5A vs. 5A2B). Notably such compound calls do not serve a suppression function insofar as male calling is not reduced compared to calling before the stimulus. These results suggest that compound calls in *K. odontotarsus* may have evolved as a form of transition call enabling advertisement calls to reduce competition as well as attract females. Moreover, the addition of suppression notes in compound calls provides females with information about both the male’s ability to attract mates and the potential suppressing effects of the male’s calls in male-male competition.

Female serrate-legged small treefrogs prefer male calls which include greater numbers of notes A and/or more note types (i.e., 2A vs. 5A, 5A vs. 5A2B). The stimuli containing more notes A (i.e. 1A, 3A, 5A) elicited more notes and more complex calls during the playback periods than were produced spontaneously before the stimuli were played back. Thus both female choice and male-male competition likely promoted the evolution of signal complexity in *K. odontotarsus*.

Sexual selection often favors the evolution of the more elaborate trait (Basolo, 1990; Ord, Blumstein & Evans, 2001; Elias, Hebets & Hoy, 2006; Akre & Ryan, 2011; Chen et al., 2012; Seddon et al., 2013), but continued trait elaboration can be inhibited if the cost entailed is high enough (Tuttle & Ryan, 1981; Endler, 1983; Bradbury & Vehrencamp, 1998; Zuk, Rotenberry & Tinghitella, 2006; Page & Ryan, 2008; Safran et al., 2008; Halfwerk et al., 2014). Thus despite the fact that female *K. odontotarsus* significantly prefer the compound call to simpler calls with only notes A (i.e., 5A vs. 5A2B), female preference does not increase when the number of B notes in compound calls is increased from two to five (i.e., 5A2B vs. 5A5B). This finding is similar to those reported in túngara frogs (Bernal et al., 2009), which suggests that female cognition limits the evolution of signal elaboration. Consistent with this idea it is unusual for males to produce five B notes in a compound call and 85.6% of all compound calls produced in natural contexts contain only one or two B notes.

Given that exaggerated and costly compound signals, such as “5A5B”, are not more attractive to females than less complex compound signals such as “5A2B”, it is important to ask why such calls would be produced at all by males. Akre and Ryan showed that the working memory of female túngara frogs favor the evolution of increasing signal complexity despite the fact that the male túngara compound call (single note whine + one or more notes chuck) production has diminishing effectiveness for both males and females beyond a single chuck (Akre & Ryan, 2010). Our results indicate that male *K. odontotarsus* produce more calls when “5A5B” was played back compared with “5A2B”, despite the fact that females do not prefer the longer call. The existence of differences in the male vocal responses evoked by playbacks of “5A2B” and “5A5B” is likely the reason why such exaggerated compound calls exist in natural contexts, and suggests that male-male competition may play an important role in the evolution of exaggerated sexual signals independently from the selective pressures imposed by female choice in *K. odontotarsus*. Our results therefore provide an alternative explanation for the evolution of exaggerated compound signals in anurans.

## Conclusions

To summarize, our results demonstrate that male-male competition and female choice have played different roles in the evolution and limitation of signal complexity in *K. odontotarsus* and show that sex differences in compound signal discrimination can serve to maintain and promote the evolution of compound signal structure. Taken together these results provide new insights into how exaggerated signals evolve and are limited in anurans.

##  Supplemental Information

10.7717/peerj.3980/supp-1 Data S1 Raw data of female phonotaxis tests

10.7717/peerj.3980/supp-2 Data S2 Raw data of male evoked vocal response experiments

## Additional Information and Declarations

### Field Study Permissions

The following information was supplied relating to field study approvals (i.e., approving body and any reference numbers): This work was conducted with the permission of the Management Office of the Mt. Diaoluo Nature Reserve.

### Data Availability

The following information was supplied regarding data availability: The raw data has been provided as Supplemental Files.
